# Supplementary material for: On-microscope staging of live cells reveals changes in the dynamics of transcriptional bursting during differentiation
Source: Nat Commun. 2022 Nov 4;13:6641. doi: 10.1038/s41467-022-33977-4 (PMC9636426; doi:10.1038/s41467-022-33977-4)
Supplement: Supplementary file 1 — Supplementary information [file 41467_2022_33977_MOESM1_ESM.pdf]

Supplementary Fig. 1

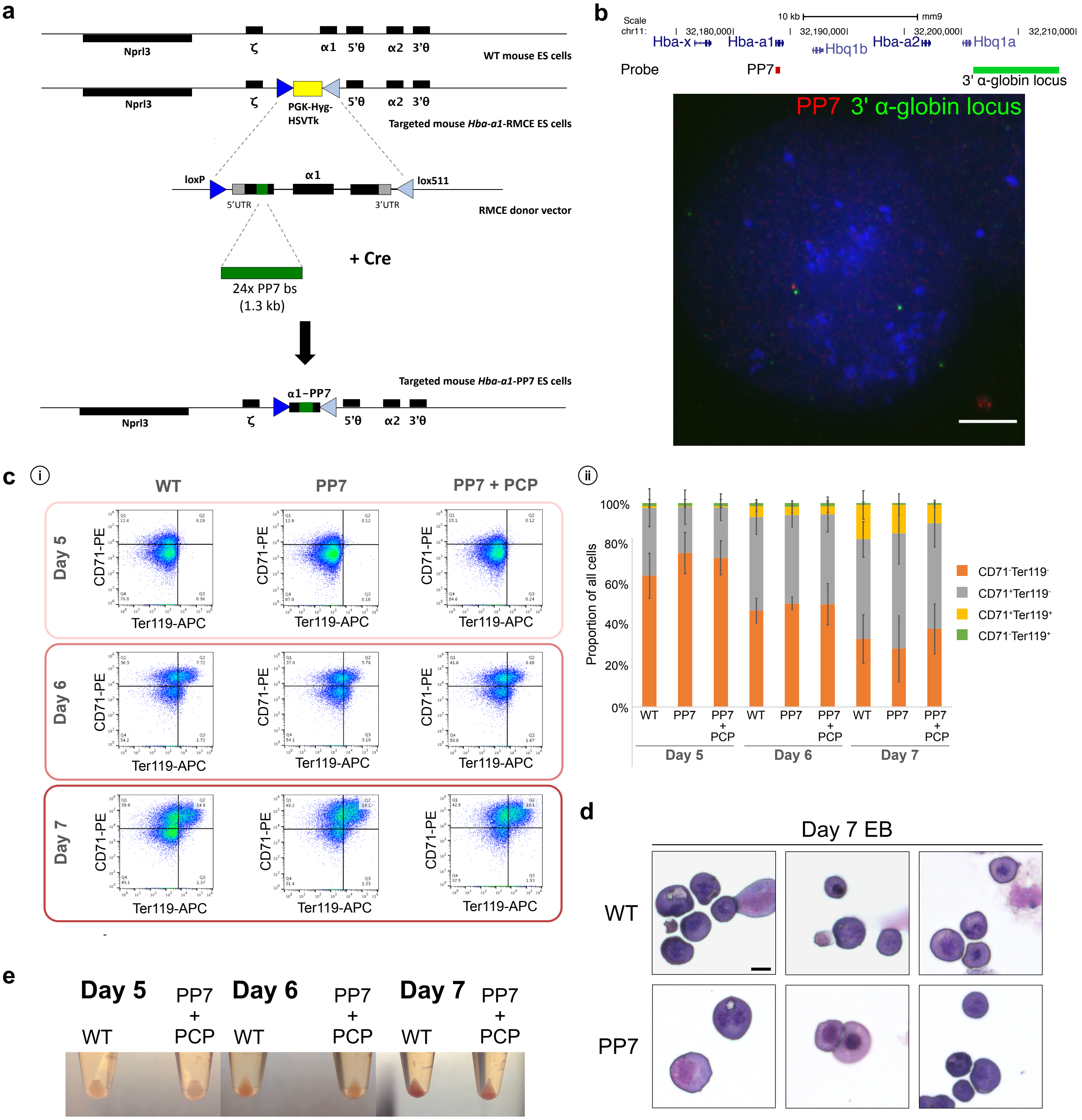

Supplementary Figure 1: Generation and characterisation of *Hba-a1*-PP7 cell line. (A) An array of 24 bacteriophage PP7 repeats was integrated into the first exon of *Hba-a1* in mouse E14 ES cells using an RMCE system. (B) Representative example of DNA FISH indicating colocalization of the PP7 repeats (red) with a probe sited at the 3' end of α-globin locus (green) in targeted mES cells. Scale = 5 µm. (C) Representative flow cytometry plots of CD71 vs Ter119 staining during EB differentiation (i) and quantification of proportion of cell population within each gate (ii). Changes in CD71 and Ter119 markers during differentiation are equivalent in WT and *Hba*-a1-PP7 (+PCP) cells. Data are presented as mean values +/- SD. n = 3 biologically independent experiments. (D) Examples of May-Grunwald-Giemsa-stained EB cells (WT and *Hba-a1*-PP7) at day 7 of differentiation. A range of erythroblast stages are observed in both cell lines. Scale = 10 µm. Similar images were obtained from three independent experiments. (E) Disaggregated EB cell pellets from WT E14 mES and *Hba-a1*-PP7 + PCP-GFP cells showing equivalent haemoglobinisation during differentiation.

Supplementary Fig. 2

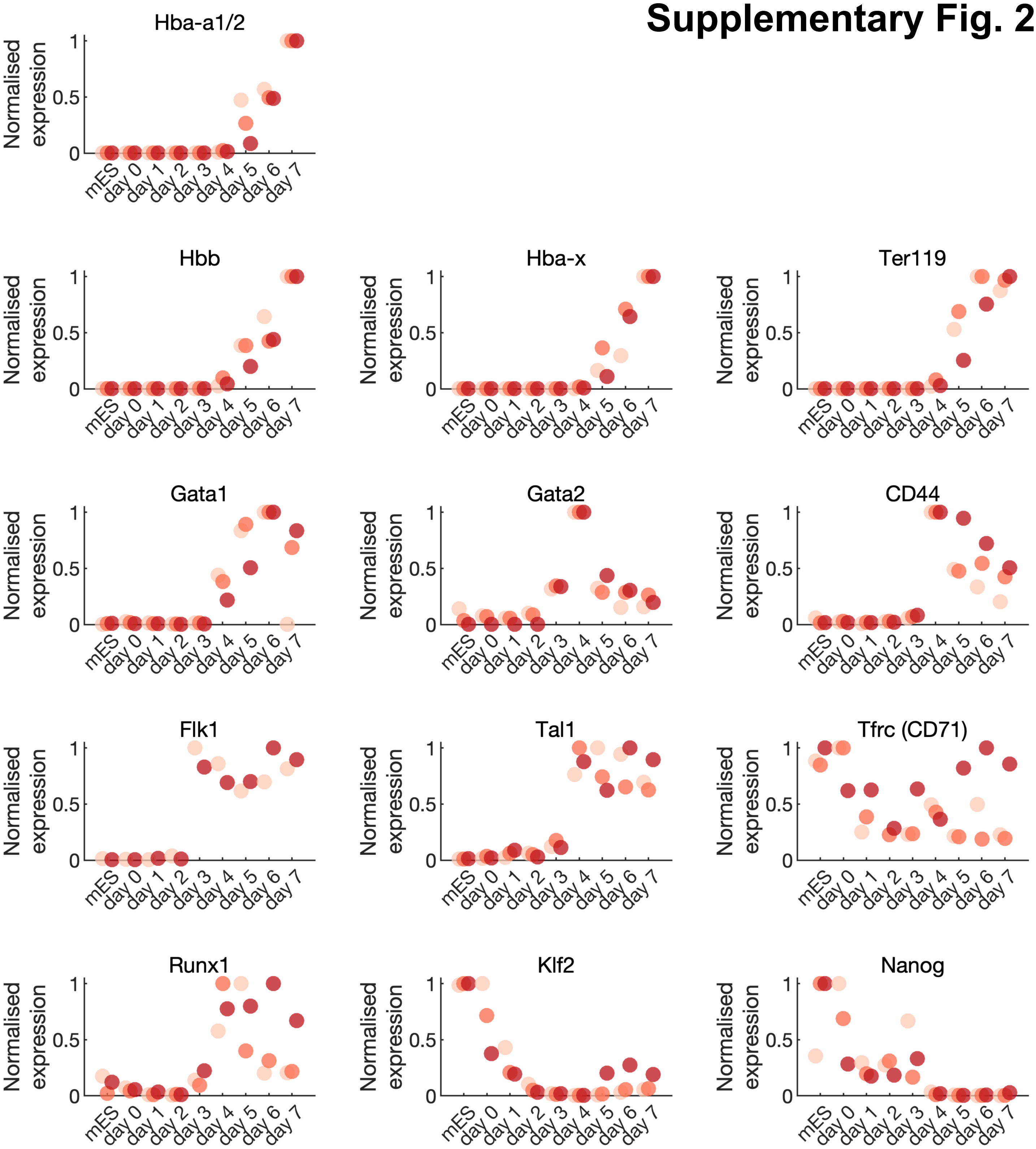

Supplementary Figure 2: RT-qPCR time course of EB differentiation from WT mES cells. A panel of stem cell and erythroid markers were profiled at each day of differentiation from mES cells to EBs. Data points from each of three experiments are shown, with colours representative of individual experiments. Data are normalised to Rn18s and then within differentiation time course for each gene. For example, the maximum value for Gata1 always occurred on day 6 of the time course and therefore day 6 values are always 1. In contrast, for Runx1 the maximum value occurred between day 4 and day 6 across different experiments leading to more variable patterns of normalised expression.

Supplementary Fig. 3

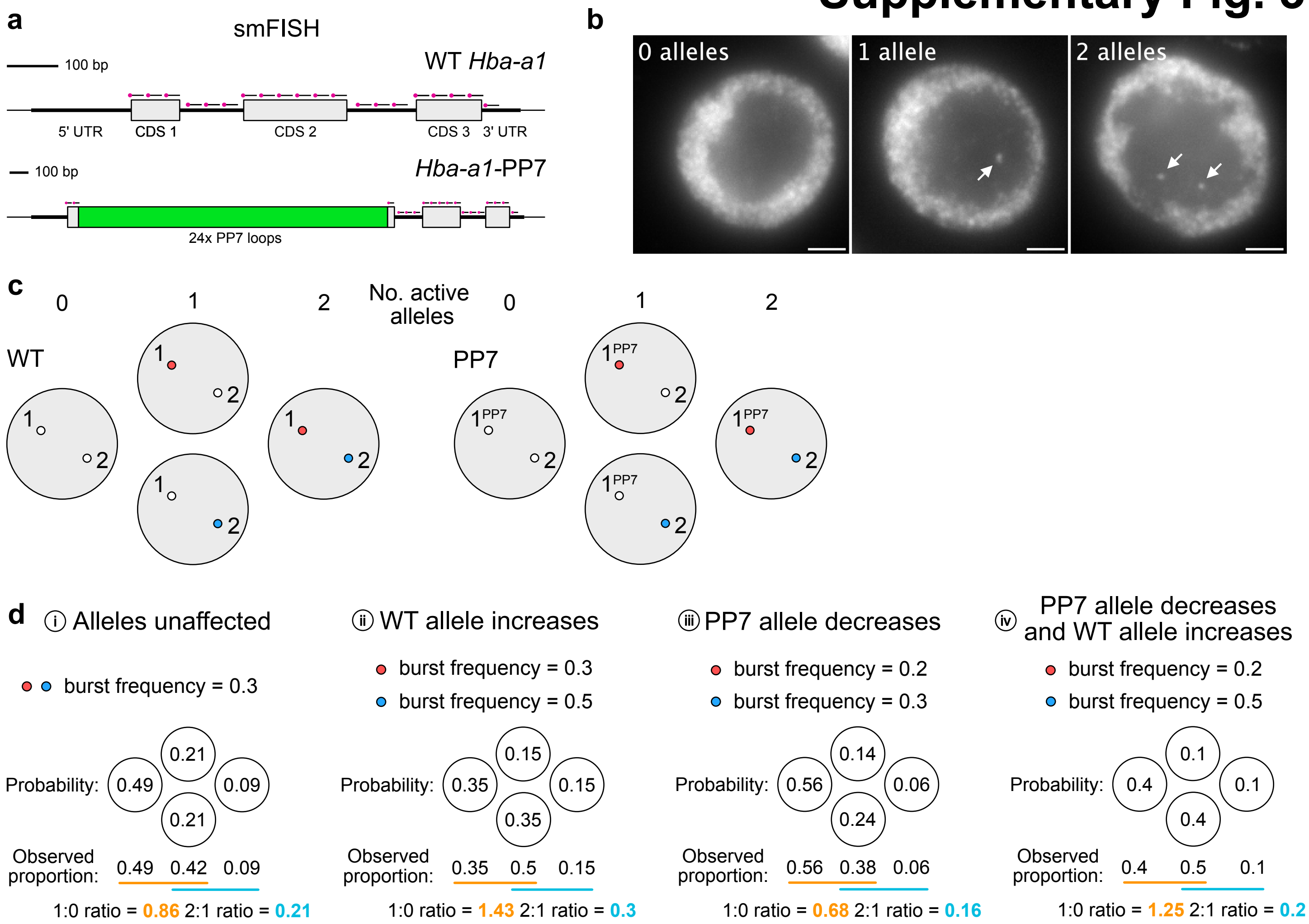

e

Genotype	Observed active alleles 0	1	2	1:0 ratio	2:1 ratio
WT	0.53	0.35	0.12	0.66	0.34
PP7	0.78	0.17	0.06	0.22	0.35
PP7 + PCP	0.65	0.26	0.09	0.4	0.35

Little change in 1:2 ratio and small decrease in 0:1 ratio = (iii) Small reduction in PP7 allele output

Supplementary Figure 3: Testing the effects of PP7 insertion on α-globin transcription at both alleles. (A) 32 smFISH probes were designed binding across the α-globin transcript and labelled with Alexa Fluor 594. These enable labelling active transcription sites for both WT and PP7 alleles. (B) Representative smFISH images showing active α-globin transcription at 0, 1 or 2 alleles in individual cells. Images are maximum intensity z-projections of seven z-slices around active foci. Scale = 2 μm. (C-D) By counting the number of active alleles by smFISH in either WT or PP7-modified cell lines we assessed the likelihood of four possible scenarios resulting from PP7 insertion: (i) there is no change in burst frequency at either allele; (ii) the WT alleles increases its output in order to compensate for loss of protein resulting from *Hba-a1*-PP7; (iii) insertion of PP7 loops causes a decrease in output at this allele with no effect at the WT allele; (iv) both alleles are affected simultaneously. Toy examples are shown, assuming each allele is activated independently of the other. The ratio between the number of active alleles observed (either 1:0 or 2:1) enables the distinction of these four possibilities. (E) The relative proportion of active alleles were measured by smFISH on WT (n=122 cells), PP7 (n=101), and PP7 cells expressing PCP-GFP (n=102). A small change 1:0 ratio and little change in 2:1 ratio suggested that there was no compensation from the WT allele with only a small reduction in output from the PP7 modified allele (see D iii). This small reduction is likely due to some polymerases falling off the longer *Hba-a1*-PP7 transgene prior to completion of the full transcript as suggested previously (Corrigan et al., 2016).

Supplementary Fig. 4

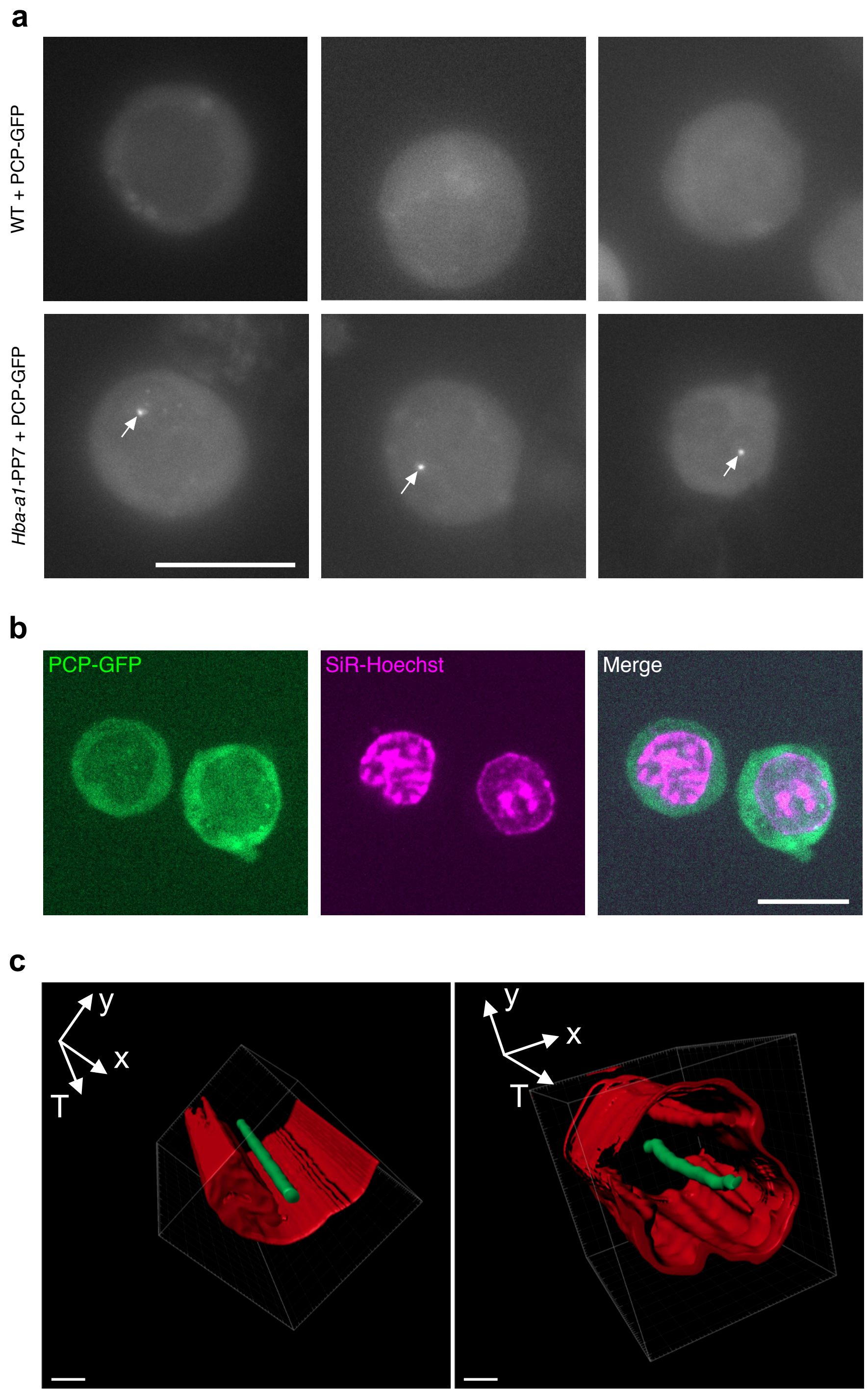

Supplementary Figure 4: Nuclear-localised foci in *Hba-a1*-PP7 cells represent sites of active transcription. (A) Examples of cells expressing PCP-GFP in WT or *Hba-a1*-PP7 clones. Bright nuclear localised foci (arrows) are only detected in cells containing PP7 loops in the *Hba-a1* gene, indicating ongoing transcriptional activity. Scale = 10 μm. (B) Co-staining of *Hba-a1*-PP7 cells with SiR-Hoechst shows depleted PCP-GFP intensity within the nucleus, enabling identification of nuclear transcription spots. Scale = 10 μm. Similar images were obtained from two independent experiments. (C) Imaris software reconstruction of representative cells over time, T, showing that bright nuclear foci (green, PCP-GFP) stay within the nucleus (red, SiR-Hoechst) for the duration of the experiment. Scale = 2 μm.

Supplementary Fig. 5

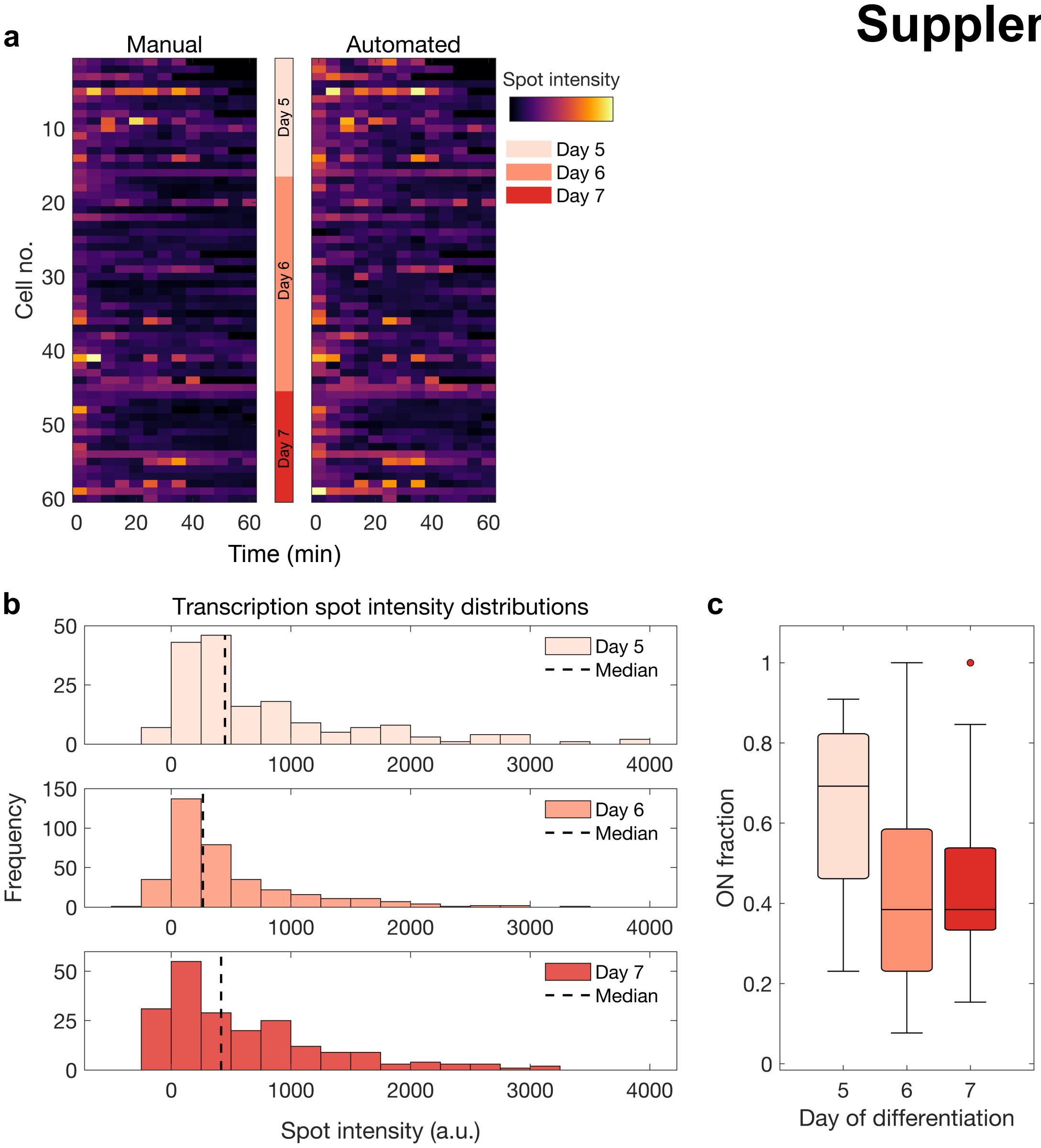

Supplementary Figure 5: α-globin transcription dynamics at day 5, 6 and 7 of EB differentiation. (A) Spot intensity traces of 60 cells from day 5, 6 and 7 of EB differentiation imaged for 1 h at 5 min frame rate. Each row represents a single cell. Cells were analysed both manually and using a semi-automated approach and the results are highly similar. (B-C) Distributions of transcription spot intensities (B) and fraction of time spent active (C) for cells cultured for 5, 6, or 7 days. Boxes within boxplots show median and interquartile range, whiskers show 9th and 91st percentile of distribution. n = 16, 29, 15 cells from day 5, 6, 7, respectively, analysed from at least three independent experiments in total.

Supplementary Fig. 6

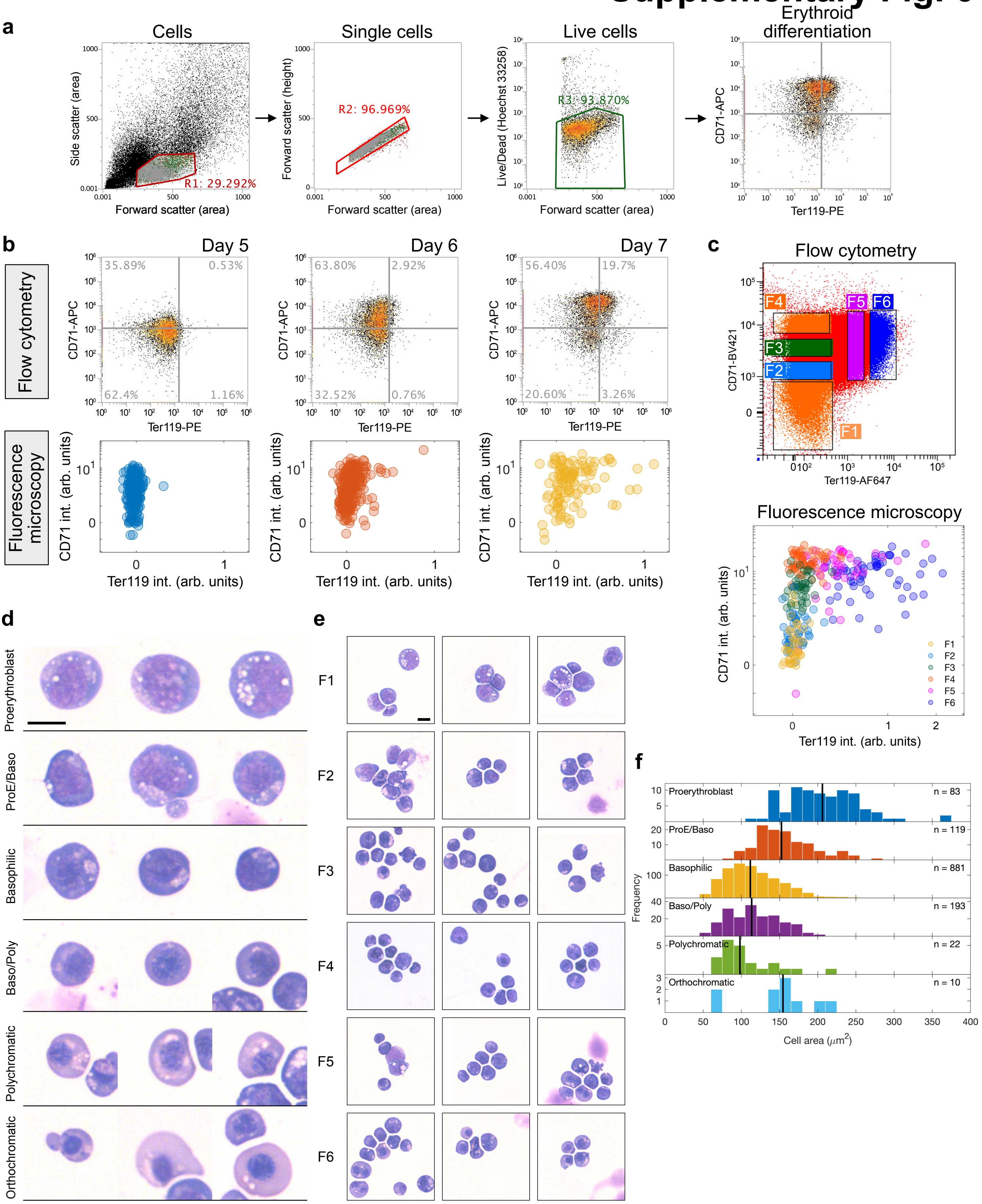

Supplementary Figure 6: Validation of on-microscope staging of EB-derived erythroid cells. (A) Flow cytometry gating strategy for profiling erythroid differentiation in EB-derived cells. (B) Comparison of CD71/Ter119 staining patterns and analysis of cells when measured by either flow cytometry or microscopy. Cells were derived from the same culture and sampled throughout a differentiation time course. (C) Day 7 EB cells were sorted into 6 populations (F1-F6) and subsequently imaged, demonstrating equivalent segregation of cells using the two modalities. (D) FACS-sorted cells from (C) were also stained with May-Grunwald-Giemsa (MGG) stain and scored according to morphology into 6 erythroblast stages, with representative example cells shown for each class. (E) Representative fields of view of MGG staining for each FACS-sorted population (F1-F6). (F) Cell area distributions for each erythroblast class. Solid line shows median cell area. n = number of cells scored. Decreasing size of progressive stages of differentiation is a well-known feature of erythropoiesis, validating our approach. Scale bars = 10 μm. The results of this experiment were consistent with all previous staging studies using this protocol.

Supplementary Fig. 7

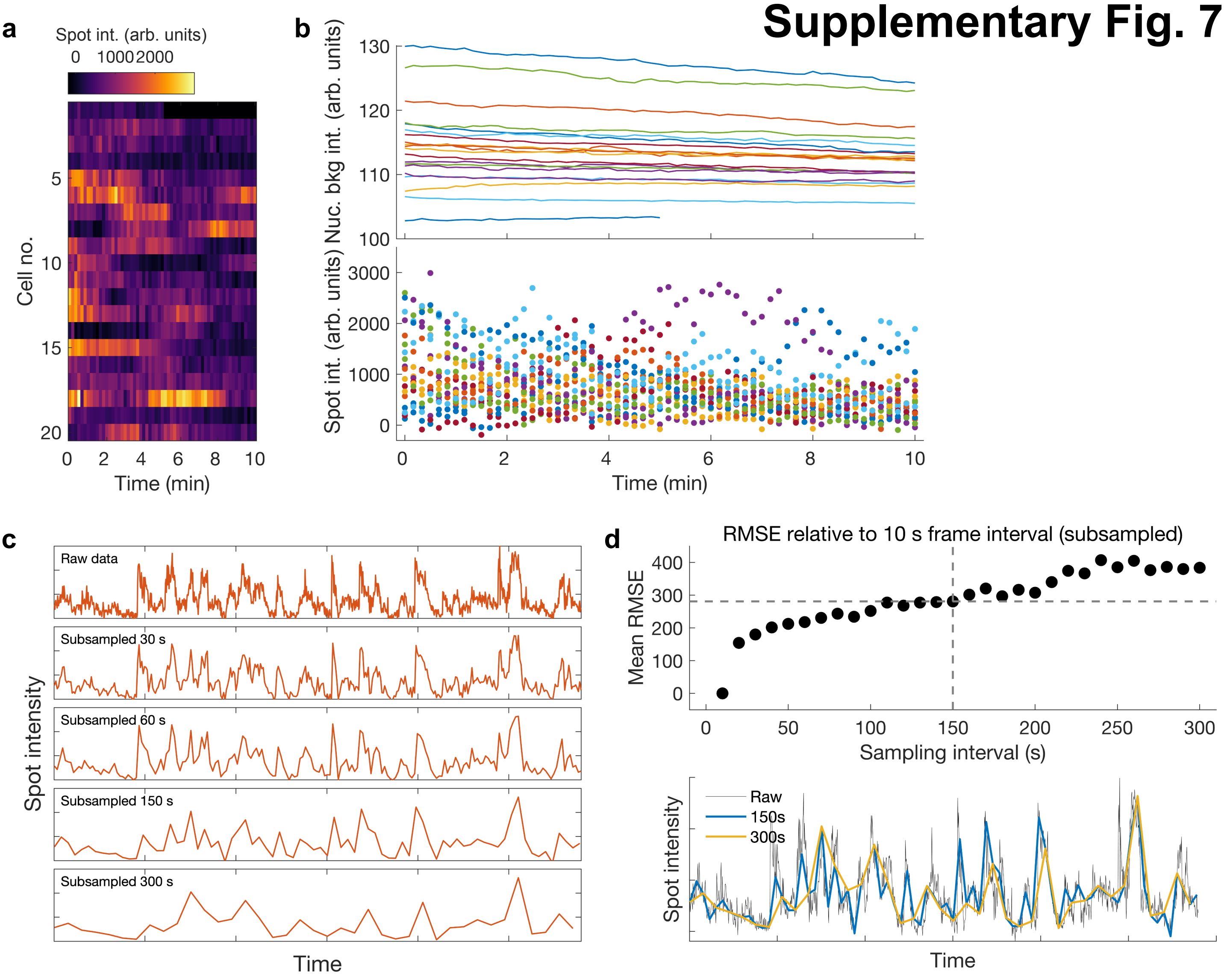

Supplementary Figure 7: Minimising photobleaching during live imaging experiments. (A) Heatmap of transcription spot intensity time series of cells imaged at high frame rate (every 10 s) for 10 min. Each row is a single cell. (B) Average nuclear background (top), and individual spot intensities (bottom) measured across image capture sessions. Different colours identify individual cells. Downward slope of overall intensity measurements during the movie indicates photobleaching and is most clearly visible after about 30 frames (5 min). (C) Raw data (top panel) from (A) was concatenated and computationally subsampled at increasing frame intervals to enable estimate of required frame rate for full capture of transcription dynamics. (D) Top panel: root mean squared error (RMSE) estimates of information lost from raw to subsampled data at increasing sampling intervals. A sampling interval of 150 s (2.5 min) represents a compromise to minimise both photobleaching and loss of dynamic transcription information. Bottom panel: visualisation of 2.5 min as an intermediate value enabling minimisation of number of frames to reduce photobleaching, while enabling capture of majority of peaks in the data, compared to 5 min sampling interval.

Supplementary Fig. 8

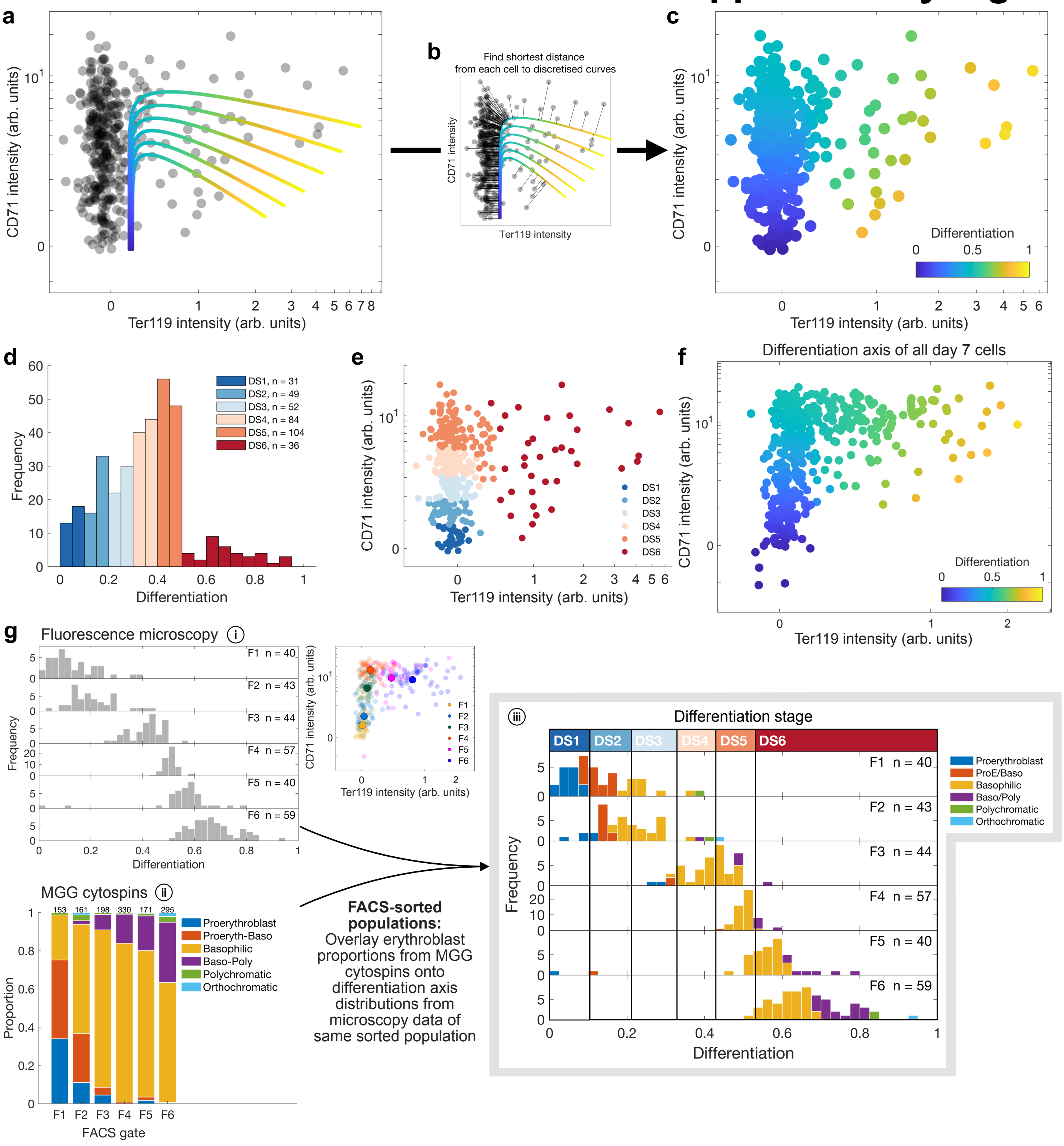

Supplementary Figure 8: Staging cells in differentiation by changes in cell surface marker intensities. (A) Multiple empirically defined curves following known changes of cell surface marker levels in differentiation ($CD71^-/Ter119^-$, $CD71^+/Ter119^-$, $CD71^+/Ter119^+$, see Supplementary Fig. 1C, 6B) were overlayed onto the CD71/Ter119 axis. (B) Each curve was discretised into individual points, and each cell was mapped to the nearest point along one of the curves. (C) The fractional distance (scaled from 0-1) of each cell along the curves was taken as a measure of differentiation progression for that cell. Blue cells are early in differentiation, orange/yellow cells are later in differentiation. (D) Histogram of position of cells along the pseudo-differentiation axis. Cells were partitioned into differentiation stages (DS1-6) according to arbitrary lengths of this differentiation axis (0.1 for DS1-DS5 and 0.5 for DS6 due to lower number of cells). (E) Cells in differentiation stages (DS1-DS6) from one-dimensional representation in D mapped back onto two-dimensional CD71/Ter119 axis, as in A. (F) As for Supplementary Fig. 8C, the position of FACS-sorted cell populations (F1-F6, Fig. 3B) along the differentiation axis was determined (blue = early differentiation, orange = late differentiation) using levels of CD71 and Ter119 intensity (as measured by fluorescence microscopy, Fig. 3B). (G) (i) For each FACS-sorted population (F1-F6), the distribution of cells along the differentiation axis (from 0-1) is shown as a histogram. The proportion of cells from the same sorted populations classified into erythroblast stages by MGG staining (ii) (as in Fig. 3D) were then overlaid onto these distributions from least to most mature (iii). The position of cells in differentiation from time-lapse imaging experiments (DS1-DS6) and FACS-sorted populations (F1-F6) could then be compared. This allowed inference of the erythroblast identity of cells in DS1-DS6. For example, DS1, at 0-0.1 on the differentiation axis, is in line with proerythroblasts (blue) across F1-F6 populations. Similarly, DS6 is a mix of late basophilic (yellow), polychromatic (purple/green) and orthochromatic (cyan) erythroblasts (~0.5-1 on the differentiation axis).

Supplementary Fig. 9

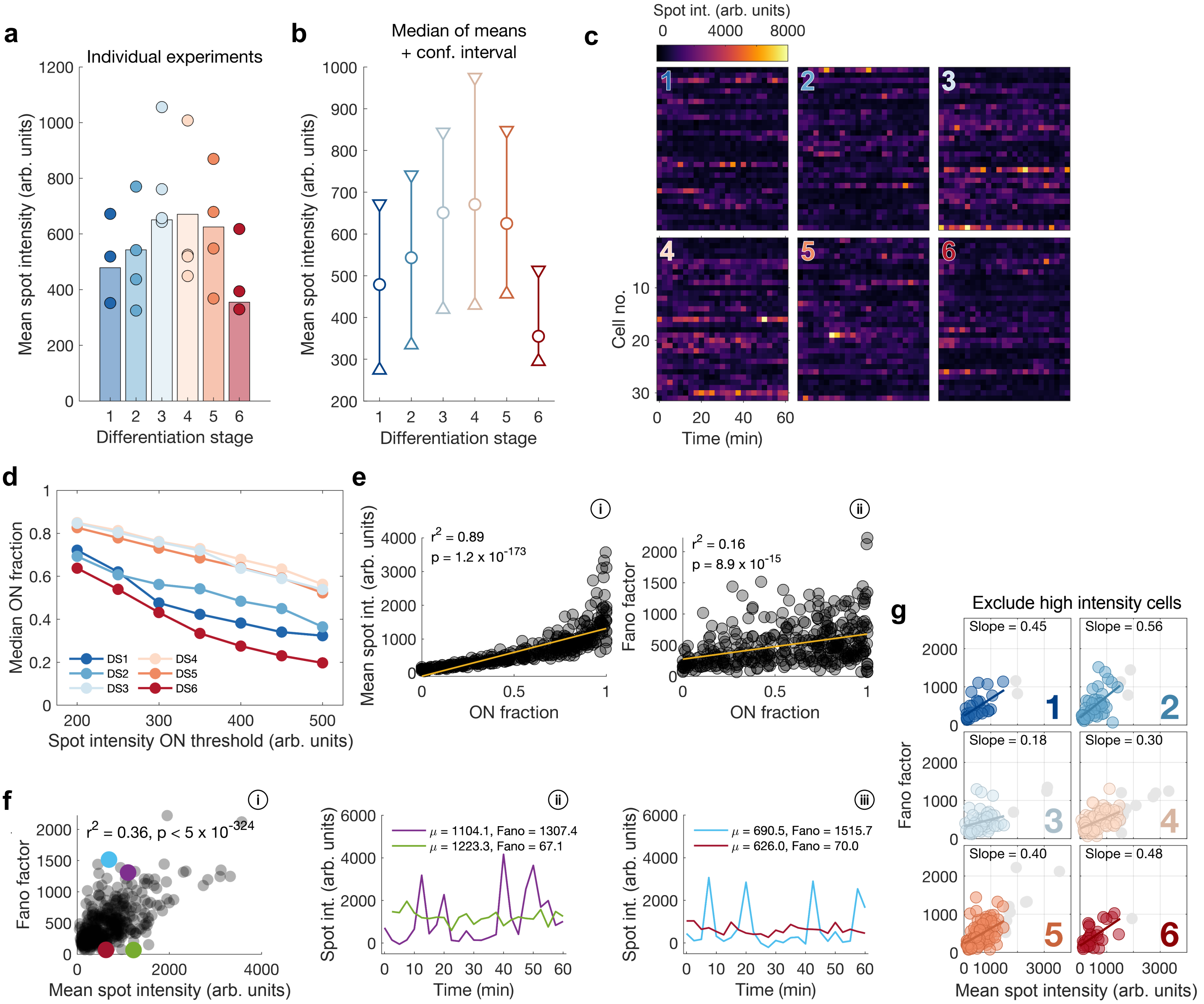

Supplementary Figure 9: Changes in α-globin transcription dynamics during erythroid differentiation. (A) Median values of mean spot intensity distributions for individual experiments (circles, where n > 5 for that differentiation stage) and all cells (bars). (B) 95% confidence intervals for the median value of the distribution of mean spot intensities for each cell. calculated by bootstrapping (random sampling with replacement) 10,000 times. Random sample contained n = 31 cells for each differentiation stage to match lowest value across stages (DS1). Observed sample medians shown as circles. (C) Heatmaps of raw spot intensity data for example individual cells across differentiation stages. (D) Median ON fraction for a range of ON/OFF thresholds for each differentiation stage. DS3-DS5 are consistently highest regardless of threshold used. (E) Spearman's rank correlation of ON fraction with mean spot intensity (i) or Fano factor (ii) for all cells. High correlation between ON fraction and mean spot intensity suggests α-globin transcriptions levels are strongly dependent on time spent transcribing. (F) Relationship between mean spot intensity and Fano factor for all cells (Spearman's rank correlation) (i), and illustrative examples of individual cell spot intensity traces (ii-iii). (G) Relationship between mean spot intensity and Fano factor across differentiation stages (DS1-DS6, as in Fig. 4E) with highly-transcribing cells removed (greyed out). The trend in the relationship across differentiation is maintained.

Supplementary Fig. 10

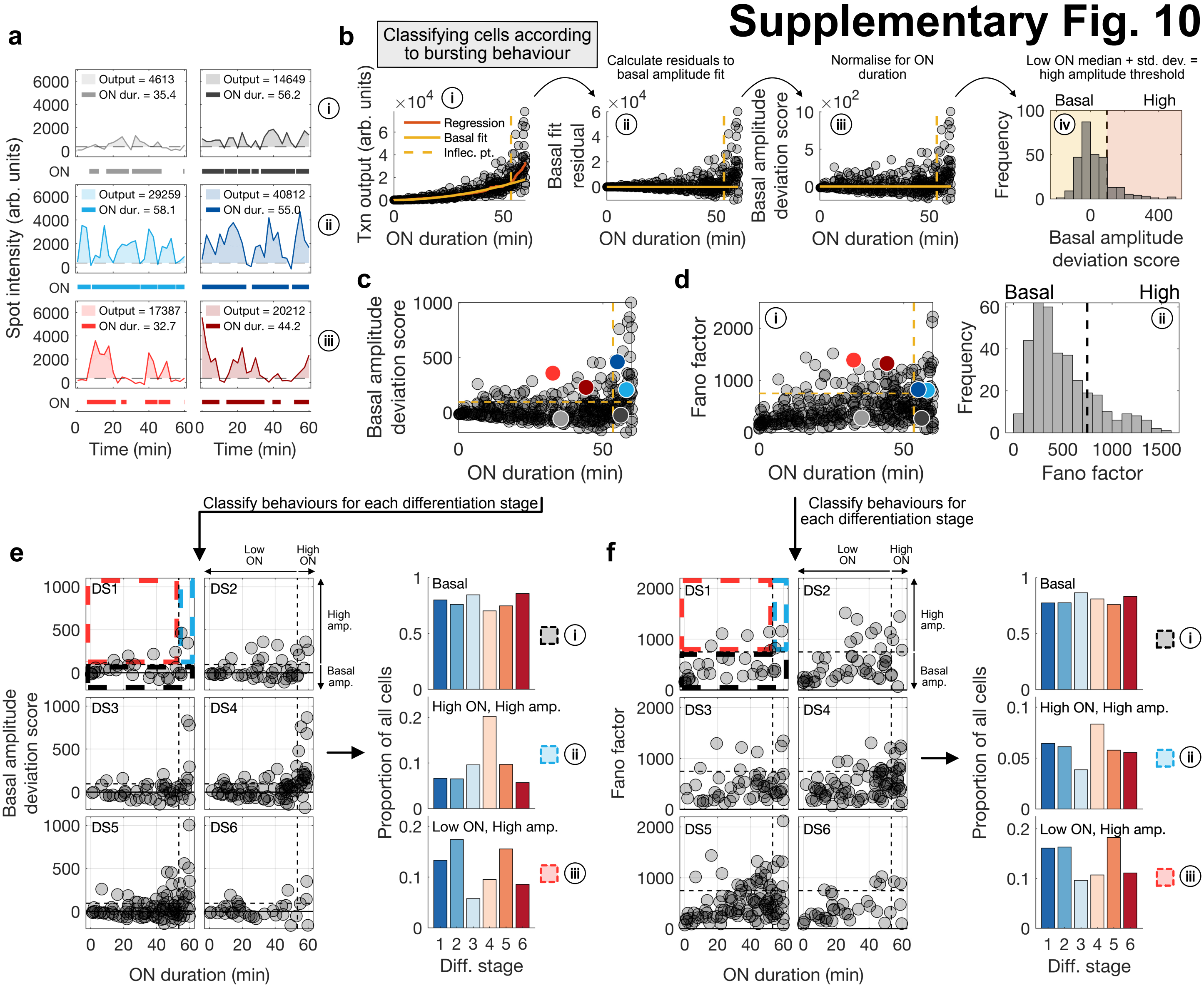

Supplementary Figure 10: Classifying dynamic behaviours of cells transcribing α-globin. (A) Example transcriptional behaviours observed across all cells: (i) 'basal amplitude' bursting, (ii) 'high ON, high amplitude' bursting, (iii) 'low ON, high amplitude' bursting (as in Fig. 5B). (B) Method for calculation of the high amplitude threshold, above which cells are said to have high average burst amplitude. The relationship between ON duration and transcriptional output (see Fig. 5A) for basal amplitude transcription was estimated by fitting a quadratic curve to local regression data points in ‘Low ON’ cells (yellow solid line, i). For each cell, residuals of transcriptional output to this basal amplitude estimate were calculated (ii) and normalised for ON duration (iii). This ‘basal amplitude deviation score’ represents the deviation of each cell from a basal amplitude bursting behaviour, i.e. if the deviation score is close to 0 then a cell is exhibiting basal amplitude bursting. ‘High amplitude’ cells are those with an deviation score of greater than one standard deviation above the median (iv). (C) Example cells from A (coloured circles) highlighted on ON duration vs basal amplitude deviation score plot. Note that the deviation score metric is able to capture the similarity in burst amplitude between example cells regardless of the ON duration. (D) (i) An equivalent analysis to C using the Fano factor instead of basal amplitude deviation score to determine whether this simpler metric could be used to segregate cells according to transcriptional behaviour. Note that the Fano factor does not capture the similarity in burst amplitude observed between example cells with different ON durations (compare red vs blue cells). (ii) The Fano factor threshold for 'high amplitude' bursting was defined, as for the basal amplitude deviation score in B, as the median plus one standard deviation. Dotted lines in C and D indicate high amplitude bursting threshold and low/high ON inflection point. (E) Basal amplitude deviation score as a function of ON duration for cells in each differentiation stage. Coloured dashed lines indicate basal amplitude cells (black), low ON high amplitude cells (red), high ON high amplitude cells (blue), demarcated by the previously defined low/high ON and basal/high amplitude thresholds. The proportion of cells in each of these parts of the plot, representing the different behaviour types, are calculated across the differentiation stages. (F) The same classification of cells by behaviour as in E, but using Fano factor instead of basal amplitude deviation score.

Supplementary Fig. 11

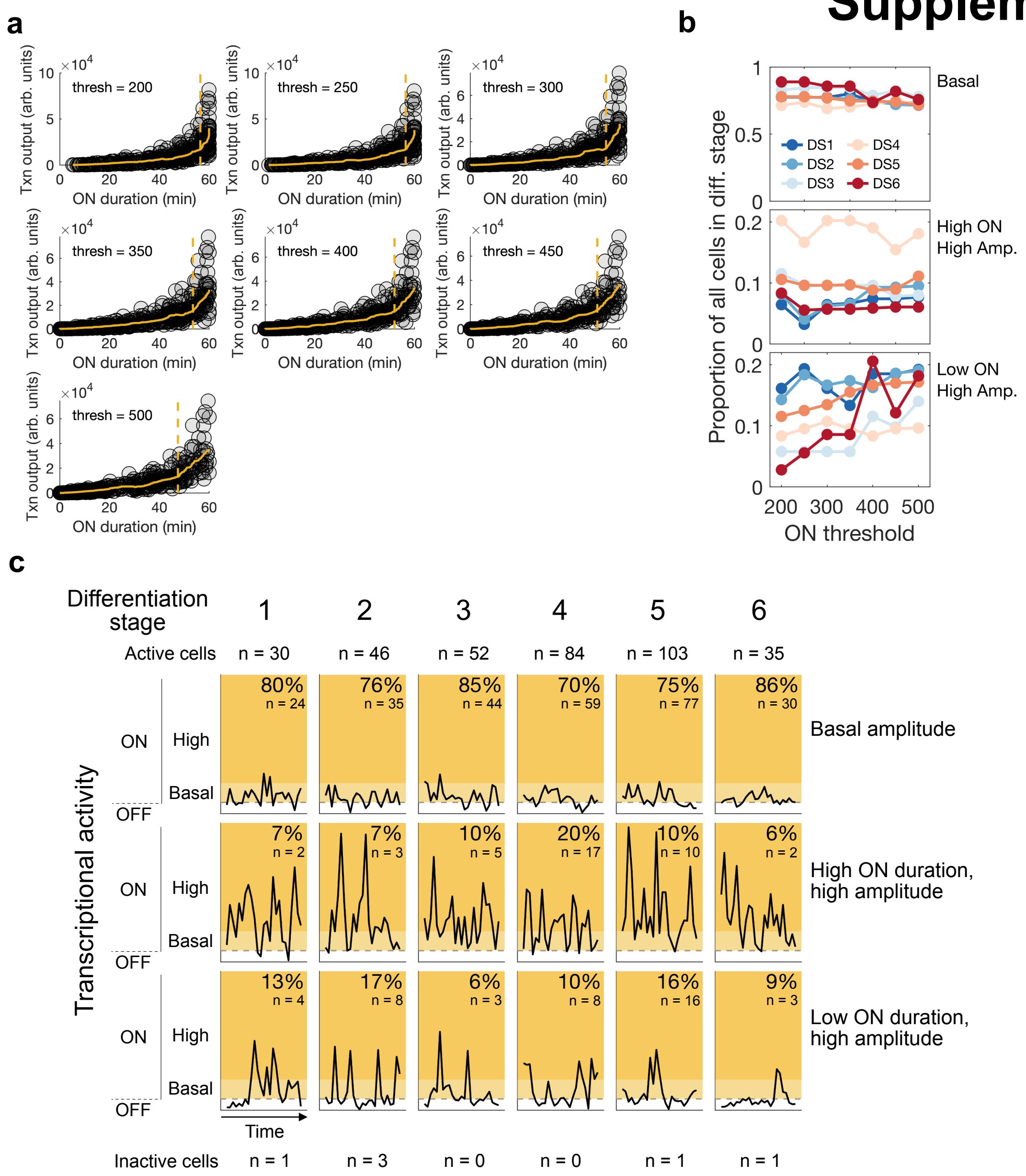

Supplementary Figure 11: Different α-globin transcriptional behaviours during differentiation. (A) Identification of Low/ High ON duration inflection point to facilitate calculation of different behaviour proportions with varying ON/OFF thresholds. (B) The proportion of different behaviours – basal amplitude transcription, high ON high amplitude transcription, low ON high amplitude transcription – throughout differentiation with varying ON/OFF threshold. ON/OFF threshold does not influence relative proportion of these behaviours at progressive differentiation stages. (C) Representative examples along with summary statistics of the number and proportions of cells with different transcriptional behaviours throughout erythropoiesis. Inactive cells are those which do not display an active transcription spot within the imaging period.

Supplementary Fig. 12

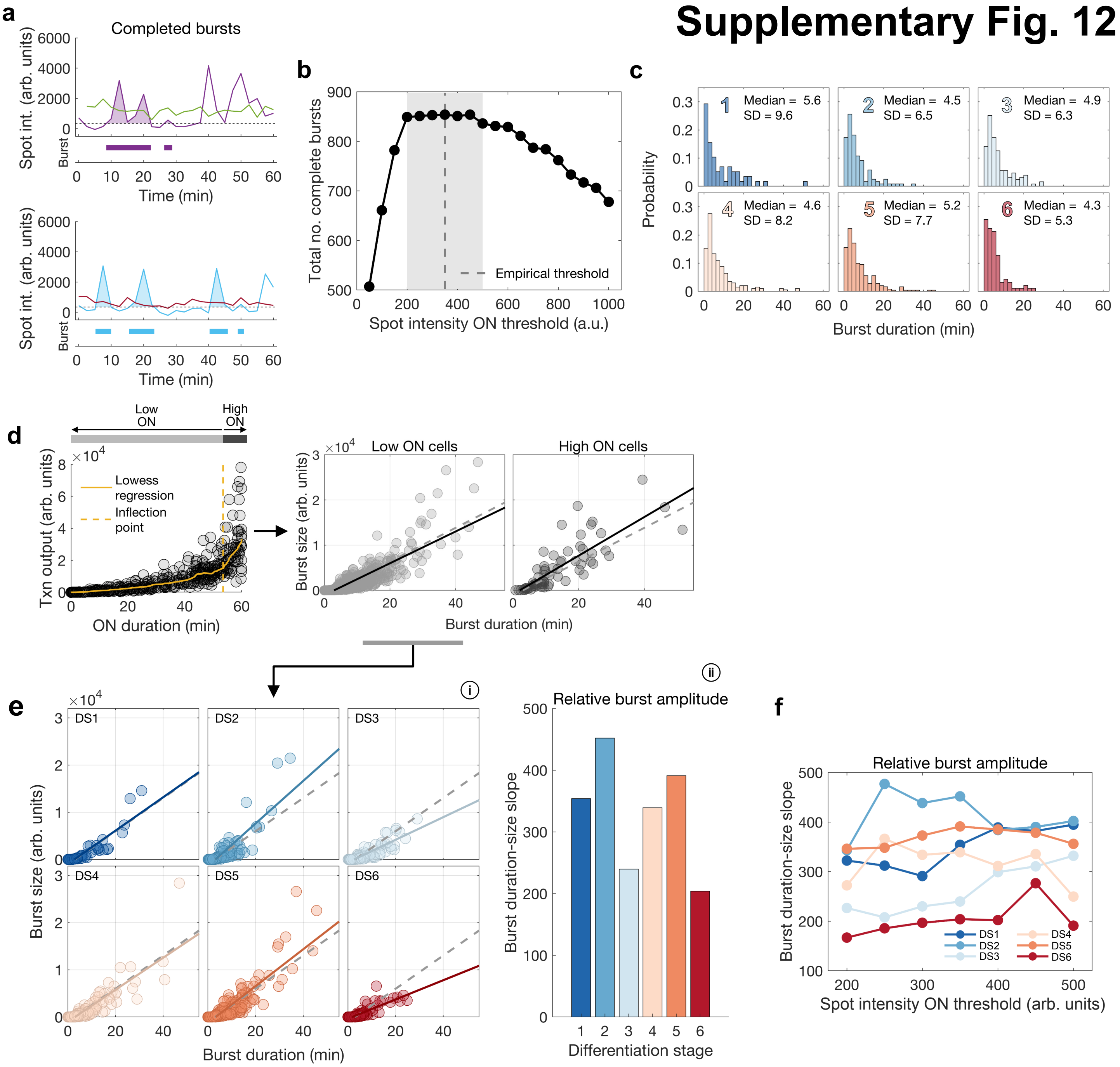

Supplementary Figure 12: Burst amplitude changes during differentiation (A) Examples of completed bursts within spot intensity traces. Only bursts which begin and end below the ON/OFF threshold are counted. Int = intensity. (B) Total number of completed bursts identified from all cells with varying ON/OFF thresholds. (C) Distributions of individual burst duration for differentiation stages 1-6. SD = standard deviation. (D) Comparison of burst duration-size relationship for low ON and high ON cells derived from Fig. 5A. Dashed line represents linear regression for all cells as reference, solid line for either low or high ON cells. High ON cells have a higher burst size for a given burst duration, and therefore a higher burst amplitude. Txn = transcriptional. (E) (i) Burst duration-size relationship for low ON cells split by differentiation stage, enabling comparison of relative burst amplitude in these cells. Solid lines are linear regression fit of cells in differentiation stages, dashed lines represent fit for all cells as a reference point. (ii) Slope of fits in (i). This represents a relative measure of burst amplitude. (F) Slope of burst duration-size relationship as in (E) with varying ON/OFF thresholds. The trends in terms of relative burst amplitude are maintained across differentiation regardless of ON/OFF threshold.

Supplementary Tables

Supplementary Table 1: Primers for RT-qPCR

Gene	Primer	Sequence
Hba-a1/2	Forward	CTGGGGAAGACAAAAGCAAC
	Reverse	GCCGTGGCTTACATCAAAGT
Hba-a1/2 (nascent)	Forward	GTGTGGATCCCGTCAACTTC
	Reverse	CCACTATGTTCCCTGCCTTG
Hba-x	Forward	CTGTCTGCTGGTCACAATGG
	Reverse	GGGAGGAGAGGGATCATAGC
Rn18s	Forward	GTAACCCGTTGAACCCCATT
	Reverse	CCATCCAATCGGTAGTAGCG
Tfrc (CD71)	Forward	TCTGGAATCCCAGCAGTTTC
	Reverse	ACCATTTGGTTGAGCTGAGG
Gypa (Ter119)	Forward	ACTCCTGTGGTGGCTTCAAC
	Reverse	TCCTCCAATGTGTGGTGAGA
Gata1	Forward	CCCAAGAAGCGAATGATTGT
	Reverse	TCCGCCAGAGTGTTGTAGTG
Gata2	Forward	GCAACCCTTACTACGCCAAC
	Reverse	GCTGTGCAACAAGTGTGGTC
Flk1	Forward	GCTTTCGGTAGTGGGATGAA
	Reverse	GGCCTTCCATTTCTGTACCA
Tal1	Forward	TCTGATGGTCCTCACACCAA
	Reverse	GTGGGGATCAGCTTTCTGAG
CD44	Forward	TGGATCCGAATTAGCTGGAC
	Reverse	AGCTTTTTCTTCTGCCCACA
Runx1	Forward	CCAGCCTCTCTGCAGAACTT
	Reverse	GACGGCAGAGTAGGGAACTG
Klf2	Forward	AACTGCGGCAAGACCTACAC
	Reverse	TCCTTCCCAGTTGCAATGAT
Nanog	Forward	CCAAAGGATGAAGTGCAAGC
	Reverse	GTGCTGAGCCCTTCTGAATC

Supplementary Table 2: Oligos for Hba-a1/2 smFISH

Hba_smFISH1	CCATGGTTTCTTCCTGAG
Hba_smFISH2	TTTGTCTTCCCCAGAGAG
Hba_smFISH3	CAGGCAGCCTTGATGTTG
Hba_smFISH4	ACCATGGCCACCAATCTT
Hba_smFISH5	GCTTCAGCTCCATATTCA
Hba_smFISH6	TCAAGGTCCTGTTCTCAC
Hba_smFISH7	AGTGCCAGGTCCATATTG
Hba_smFISH8	TTAGAAGCTGCCCACTGA
Hba_smFISH9	TGGTGGGGAAGCTAGCAA
Hba_smFISH10	GTGAGGGAAGTAGGTCTT
Hba_smFISH11	AGAGCCGTGGCTTACATC
Hba_smFISH12	CGACCTTCTTGCCGTGAC
Hba_smFISH13	AGCATTGGCCAGAGCATC
Hba_smFISH14	GGCAGGTCATCGAGGTGG
Hba_smFISH15	TCAGAGCAGACAGGGCA
Hba_smFISH16	CTTGTGGGCATGCAGGTC
Hba_smFISH17	TTGACGGGATCCACACGC
Hba_smFISH18	TCCCAGCGCATACCTTGA
Hba_smFISH19	TCCTAGGGGTCCCAGATG
Hba_smFISH20	TGCCTTGGGCACGAGGAC
Hba_smFISH21	CTTCCTGGGACCACTATG
Hba_smFISH22	AAGTGGACACCCTGATGC
Hba_smFISH23	CAGGCAGTGGCTCAGGAG
Hba_smFISH24	GTGGCTAGCCAAGGTCAC
Hba_smFISH25	CGGGGGTGAAATCGGCAG
Hba_smFISH26	TGTCCAGAGAGGCATGCA
Hba_smFISH27	GCTCACAGAGGCAAGGAA
Hba_smFISH28	TACTTGGAGGTCAGCACG
Hba_smFISH29	CGCAGAAGGCAGCTTAAC
Hba_smFISH30	CATGGCCAGAAGGCAAGC
Hba_smFISH31	ACCAAGAGGTACAGGTGC
Hba_smFISH32	TCTTCCTACTCAGGCTTT

Supplementary Notes

Classification of transcriptional behaviours from time series data

Using summary statistics such as the mean and Fano factor we noticed differences in the distributions of these metrics as cells progress through erythropoiesis. To explore this further we measured the total time spent in the active state (ON duration) for each cell and compared this to the total transcriptional output (defined by the area under the curve between the ON threshold as a lower bound, and the fluctuating spot intensity as an upper bound).

In general, when comparing the relationship between these two variables we observed that the majority of cells are found along a baseline trajectory where total transcriptional output slowly increases as ON duration increases, suggesting these cells are predominantly transcribing at a low, basal rate (Fig. 5A, B). To describe the 'average' relationship between ON duration and transcriptional output we used local regression. This nicely captured this basal behaviour of most cells but at high ON duration the gradient of the regression showed a marked increase, with a clear inflection point at around 53.5 min ON duration (Fig. 5A). This suggested that on average the burst amplitude in near-continuously active cells is higher than those which are actively only intermittently, since the gradient of the regression depends on the relative changes in total transcriptional output (and therefore average burst amplitude) with increased ON duration. Given the fact that Dar et al. (2012) previously described a similar phenomenon where burst size (of which amplitude is a component) is increased only beyond a particular threshold at high levels of expression, we wanted to classify cells according to this change in bursting behavior. Therefore, we used this inflection point to classify cells as either 'high ON' (active > 53.5 min, > 89% of the time) or 'low ON' (active < 53.5 min).

We then wanted to classify cells into those exhibiting largely basal amplitude bursts, and those transcribing with high amplitude bursts, at either low or high ON durations (Fig. 5B, Supplementary Fig. 10A). To do this, we first extrapolated the trajectory of basal amplitude bursting cells using a simple quadratic fit of the LOWESS regression itself in low ON cells only (Supplementary Fig. 10Bi).

We then calculated the residual of each cell to this extrapolated fit to enable quantification of the number of cells which lie significantly above the basal amplitude duration-vs-output trajectory (Supplementary Fig. 10Bii). Given that those with higher ON duration have a greater chance of having sufficiently increased transcriptional output to lie above this trajectory (simply due to having been active for longer) we normalized these residuals by the ON duration (Supplementary Fig. 10Biii). We called this metric the 'basal amplitude deviation score' which essentially measures the extent to which the bursting profile of an individual cell differs from a 'basal amplitude' bursting behaviour. We then defined a threshold above which cells would be classified as 'high amplitude' bursting cells using the distribution of these normalized residuals. Specifically, we defined the threshold as one standard deviation away from the median of normalized residuals at low ON duration (Supplementary Fig. 10Biv).

We also checked whether, instead of using our basal amplitude deviation score for classifying cell behaviours according to burst amplitude, we could use the Fano factor as a simpler alternative to do this. Similar to the above analysis (Supplementary Fig. 10C) we visualized the relationship between ON duration and Fano factor (Supplementary Fig. 10D), defined a threshold above which cells might be classified as 'high amplitude' bursting (median plus standard deviation of Fano factor distribution across all cells) and split cells into the same classes as before – 'basal', 'high ON, high amplitude', 'low ON, high amplitude'. However, we observed that cells with similar levels of high amplitude bursting, for example, were not similarly captured using the Fano factor analysis as compared to the basal amplitude deviation score approach. For either of these to be a good metric to use they must be able to identify high amplitude cells regardless of the total time spent transcribing (i.e. either 'high ON' or 'low ON'). Cells with 'high ON, high amplitude' bursting had reduced Fano factor compared to 'low ON, high amplitude' bursting (compare red and blue example cells in Supplementary Fig. 10D). This is in contrast to the basal amplitude deviation score analysis, where cells with similarly high amplitude bursts have similar deviation scores regardless of ON duration (Supplementary Fig. 10C). As a result, while the proportion of cells in the 'basal' and 'low ON, high amplitude' categories across differentiation were similar to the deviation score analyses, 'high ON, high amplitude' cells were under-represented at all differentiation stages and did not show the same peak in mid stages of differentiation as with the basal amplitude deviation score comparison.

Considering all of the above, we concluded that the use of the basal amplitude deviation score is preferable to the Fano factor for dissecting the behaviour of cells according to their burst amplitude, and therefore this data is included in Figure 5C.

To enable us to check that our analysis was not affected by the empirically defined threshold for when a gene is called as active or ON we repeated the analysis with different values for this threshold (Supplementary Fig. 11A, B). The inflection point was manually identified in each case according to the LOWESS regression, as before. Little change was observed in the relative proportions of cells exhibiting different transcriptional behaviours across the differentiation stages using this approach, suggesting these observations are robust to variation in the spot intensity ON threshold (Supplementary Fig. 11B).

Benefits of on-microscope staining for staging cells in differentiation

In this study we exploit a simple on-microscope staining approach[2] to enable us to analyse cells across a range of differentiation states. The benefits of such an approach are several-fold. Firstly, the sensitivity of nascent transcriptional activity to environmental stimuli[3–7] illustrates the need for gentle experimental practices when studying transcription. Popular methods for isolation of immunophenotyped cells such as magnetic bead selection can induce a stress response, with FACS having a somewhat lesser effect[8]. On-microscope labelling therefore represents an attractive alternative by reducing the mechanical stimulation of cells. Secondly, given the time required for the above isolation methods (1-4 h depending on the rarity of cell populations) as well as the need to allow cells to acclimatise (30 min-1 h) before imaging nascent transcription, our approach offers significant time savings depending on the number of populations to be imaged. Finally, physiological changes in development, including transcription, frequently occur along a continuum of cell states. In contrast to the other methods mentioned above, our approach enables simultaneous capture of transcription dynamics across this developmental spectrum without the need for arbitrary grouping of cells before the experiment takes place. One limitation of this method is the requirement for stratification of differentiation stages with only a few cell surface markers (given the limits on number of imaging channels for live cell confocal microscopy). However, we believe there are numerous examples of differentiation programmes for which this should not be a hindrance, such as early

T cell development[9,10], or during reprogramming of human pluripotent stem cells to a naïve state[11].

Supplementary References

1. Dar, R. D. *et al.* Transcriptional burst frequency and burst size are equally modulated across the human genome. *Proceedings of the National Academy of Sciences* 109, 17454–17459 (2012).

2. Eilken, H. M., Nishikawa, S.-I. & Schroeder, T. Continuous single-cell imaging of blood generation from haemogenic endothelium. *Nature* 457, 896–900 (2009).

3. Cesbron, F., Oehler, M., Ha, N., Sancar, G. & Brunner, M. Transcriptional refractoriness is dependent on core promoter architecture. *Nat Commun* 6, 6753 (2015).

4. Falo-Sanjuan, J., Lammers, N. C., Garcia, H. G. & Bray, S. J. Enhancer Priming Enables Fast and Sustained Transcriptional Responses to Notch Signaling. *Dev. Cell* 50, 411-425.e8 (2019).

5. Kindgren, P., Ivanov, M. & Marquardt, S. Native elongation transcript sequencing reveals temperature dependent dynamics of nascent RNAPII transcription in Arabidopsis. *Nucleic Acids Res.* 48, 2332–2347 (2019).

6. Mahat, D. B., Salamanca, H. H., Duarte, F. M., Danko, C. G. & Lis, J. T. Mammalian Heat Shock Response and Mechanisms Underlying Its Genome-wide Transcriptional Regulation. *Mol. Cell* 62, 63–78 (2016).

7. Stavreva, D. A. *et al.* Transcriptional Bursting and Co-bursting Regulation by Steroid Hormone Release Pattern and Transcription Factor Mobility. *Mol. Cell* 75, 1161-1177.e11 (2019).

8. Beliakova-Bethell, N. *et al.* The effect of cell subset isolation method on gene expression in leukocytes. *Cytometry A* 85, 94–104 (2014).

9. Kernfeld, E. M. *et al.* A Single-Cell Transcriptomic Atlas of Thymus Organogenesis Resolves Cell Types and Developmental Maturation. *Immunity* 48, 1258-1270.e6 (2018).

10. Shukla, S. *et al.* Progenitor T-cell differentiation from hematopoietic stem cells using Delta-like-4 and VCAM-1. *Nat. Methods* 14, 531–538 (2017).

11. Bredenkamp, N., Stirparo, G. G., Nichols, J., Smith, A. & Guo, G. The Cell-Surface Marker Sushi Containing Domain 2 Facilitates Establishment of Human Naive Pluripotent Stem Cells. *Stem Cell Reports* 12, 1212–1222 (2019).
