# Supplementary material for: On-microscope staging of live cells reveals changes in the dynamics of transcriptional bursting during differentiation
Source: Nat Commun. 2022 Nov 4;13:6641. doi: 10.1038/s41467-022-33977-4 (PMC9636426; doi:10.1038/s41467-022-33977-4)
Supplement: Supplementary file 3 — Description of additional Supplementary File [file 41467_2022_33977_MOESM3_ESM.pdf]

Descriptions of Additional Supplemental Files

Supplementary Movies 1-4: (A) Example movie of EB-derived *Hba-a1*-PP7 erythroid cell from day 6 of differentiation. Red circle indicates location of transcription spot over time. (B) Quantification of spot intensity from A. (C) Maximum projected stacks of anti-CD71 Brilliant Violet 421 and anti-Ter119 Alexa Fluor 647 staining of example cell, as well as PCP-GFP signal. (D) Quantification of fluorescence intensity of CD71 and Ter119 levels from C (red circle) compared to all cells within the population.

Supplementary Software 1: File required to fully recapitulate plots of FAC-sorted cell populations subsequently imaged on the microscope. CD71/Ter119 intensities from imaged cells are presented on a biexponential scale (as for flow cytometry data) which was achieved here using the 'logicleTransform' function in Matlab. The result of using this transform is that specific axes labels are required to properly present the data. This file enables full recapitulation of the plots in the manuscript by providing these custom axes labels.
